# The Genomics of Speciation in *Drosophila*: Diversity, Divergence, and Introgression Estimated Using Low-Coverage Genome Sequencing

**DOI:** 10.1371/journal.pgen.1000550

**Published:** 2009-07-03

**Authors:** Rob J. Kulathinal, Laurie S. Stevison, Mohamed A. F. Noor

**Affiliations:** 1 Department of Molecular and Cellular Biology, Harvard University, Cambridge, Massachusetts, United States of America; 2 Biology Department, Duke University, Durham, North Carolina, United States of America; University of Arizona, United States of America

## Abstract

In nature, closely related species may hybridize while still retaining their distinctive identities. Chromosomal regions that experience reduced recombination in hybrids, such as within inversions, have been hypothesized to contribute to the maintenance of species integrity. Here, we examine genomic sequences from closely related fruit fly taxa of the *Drosophila pseudoobscura* subgroup to reconstruct their evolutionary histories and past patterns of genic exchange. Partial genomic assemblies were generated from two subspecies of *Drosophila pseudoobscura* (*D. ps.*) and an outgroup species, *D. miranda*. These new assemblies were compared to available assemblies of *D. ps. pseudoobscura* and *D. persimilis*, two species with overlapping ranges in western North America. Within inverted regions, nucleotide divergence among each pair of the three species is comparable, whereas divergence between *D. ps. pseudoobscura* and *D. persimilis* in non-inverted regions is much lower and closer to levels of intraspecific variation. Using molecular markers flanking each of the major chromosomal inversions, we identify strong crossover suppression in F_1_ hybrids extending over 2 megabase pairs (Mbp) beyond the inversion breakpoints. These regions of crossover suppression also exhibit the high nucleotide divergence associated with inverted regions. Finally, by comparison to a geographically isolated subspecies, *D. ps. bogotana*, our results suggest that autosomal gene exchange between the North American species, *D. ps. pseudoobscura* and *D. persimilis*, occurred since the split of the subspecies, likely within the last 200,000 years. We conclude that chromosomal rearrangements have been vital to the ongoing persistence of these species despite recent hybridization. Our study serves as a proof-of-principle on how whole genome sequencing can be applied to formulate and test hypotheses about species formation in lesser-known non-model systems.

## Author Summary

The transformation of populations into distinct species depends on whether hybridization, recombination, and subsequent gene introgression can be suppressed between diverging species. We use partial genome sequences to reconstruct this evolutionary process in the *Drosophila pseudoobscura* species subgroup, which includes the hybridizing species pair *D. pseudoobscura pseudoobscura* and *D. persimilis*. Recent models suggest that chromosomal inversions can facilitate the persistence of hybridizing species because of their effects on recombination, whereby inverted regions would exhibit higher nucleotide divergence than non-inverted regions. Indeed, *D. pseudoobscura-D. persimilis* nucleotide divergence outside these inverted regions is lower than within or near inversions, resembling *D. ps. pseudoobscura* levels of within-species nucleotide diversity. We also observe that recombination suppression in F_1_ hybrids extends greater than 2 Mbp outside the inversion breakpoints. Furthermore, when genomic sequence of *D. persimilis* is compared to two sister subspecies—the hybridizing subspecies, *D. ps. pseudoobscura*, and a non-hybridizing control subspecies, *D. ps. bogotana*—autosomal divergence is lower in the former, demonstrating recent gene exchange. These lines of evidence support a speciation model in which the two hybridizing species persist despite the presence of recent genic introgression in collinear regions of the genome because of the reduced recombinational effects of the inversions that distinguish them.

## Introduction

One of the most significant empirical insights in evolutionary biology is that a large number of species naturally hybridize with close relatives [see reviews in 1],[2], and these species pairs often exchange genetic material (“introgression”). These insights contrast previous assertions that considered interspecies hybridization as rare or anomalous [e.g., 3]. However, recognizing that hybridization and introgression are frequent suggests that genetic features may allow such species to remain distinct.

Several recent studies suggested that genomic regions of low recombination may provide a means to create “islands of differentiation” between species [4]–[10]. While introgression may homogenize parts of genomes, regions of low recombination in hybrids, such as those within chromosomal inversions, maintain their distinction despite the influx of foreign alleles in collinear regions. This pattern of localized differentiation should be particularly strong if regions of low recombination also harbor loci with divergently selected alleles or alleles conferring reproductive isolation. However, this hypothesis has been difficult to test rigorously on a genome-wide scale.

Assembled whole genome sequences and inexpensive resequencing technologies can complement locus-specific sequencing studies and genetic mapping studies for clarifying the role of regions of low recombination in species persistence. Fruit flies from the genus Drosophila have been a major focus of many studies of nucleotide divergence between closely related species and mapping studies of traits that prevent introgression such as hybrid sterility. The recent sequencing of multiple closely related Drosophila species [11],[12] places us in a stronger position to test hypotheses concerning gene flow and speciation in this model system.

### A Speciation Model System: The *Drosophila pseudoobscura* Species Subgroup

The *Drosophila pseudoobscura* species subgroup is comprised of two *D. pseudoobscura* subspecies (*D. ps. pseudoobscura* and *D. ps. bogotana*), and two closely related species, *D. persimilis* and *D. miranda*. The *D. pseudoobscura* subspecies are geographically isolated (*D. ps. pseudoobscura* ranges across the western half of North America and *D. ps. bogotana* is restricted to Colombia in South America), share chromosomal arrangements, and represent the earliest stages of species divergence [13]. *D. persimilis* and *D. miranda* are restricted to the west coast of North America, where they co-occur with *D. ps. pseudoobscura*. Both *D. pseudoobscura* subspecies differ from the close relative species *D. persimilis* by fixed (or nearly fixed) chromosomal inversion differences on three of their major chromosome arms, and F_1_ hybrid males from crosses between these species are sterile (though females are fertile). In contrast, *D. miranda* is an outgroup species which cannot produce any fertile hybrids with *D. pseudoobscura* or *D. persimilis* [14]. The relative relationships of these species as (((*D. ps. pseudoobscura-D. ps. bogotana*) *D. persimilis*) *D. miranda*) is well established by DNA sequences, chromosomal inversions, and reproductive isolation [15],[16]. Overall, this system provides us with a pair of taxa that hybridize and have experienced introgression (*D. ps. pseudoobscura* and *D. persimilis* [17]–[19]), and two taxa that have not experienced recent introgression from any close relatives (ingroup, *D. ps. bogotana*, and outgroup, *D. miranda*).

Nucleotide divergence between the hybridizing species *D. ps. pseudoobscura* and *D. persimilis* is high within and near the three chromosomal inversions [20],[21], which are linked to factors conferring hybrid sterility, mating discrimination, and other barriers to gene flow [22],[23]. Based on these observations, we hypothesized that inversions facilitate the distinction of these species despite ongoing natural hybridization. However, it has been difficult to fully disentangle complications that result from ancestral polymorphisms shared between these species [21],[24] and underlying assumptions found in many statistical tests for introgression [25]–[27]. Two recent studies also reached differing conclusions about whether gene exchange between these species occurred during the initial divergence process or later [20],[21].

To better understand the genealogical history of this subgroup, we use published genome sequence assemblies of *D. ps. pseudoobscura and D. persimilis* [12],[28], along with three novel partial genomic sequences that we generated using 454/Roche technology (Table S1): one from *D. miranda*, one from a second strain of the North American subspecies, *D. ps. pseudoobscura*, and one from the South American subspecies, *D. ps. bogotana*. By providing controls for divergence in the absence of gene flow, these newly obtained genomic sequences allow for more robust analysis than previous studies. Our new results suggest that the chromosomal regions inverted between *D. persimilis* and *D. ps. pseudoobscura* arose in allopatry, and that *D. pseudoobscura, D. persimilis*, and *D. miranda* all diverged within a relatively short time frame. We also find compelling evidence for autosomal gene exchange between *D. persimilis* and *D. ps. pseudoobscura* in collinear regions since the split of *D. ps. pseudoobscura* and *D. ps. bogotana*, likely within the past 200,000 years. Overall, our analyses utilize genome sequence data in an existing framework to demonstrate the importance of chromosomal inversions in maintaining the persistence of hybridizing species and to consolidate previous tentative conclusions about divergence in this group. Further, this research serves as a model for how whole genome shotgun sequence data can be used with a reference genome sequence to address fundamental questions regarding evolutionary changes leading to the formation of species.

## Results

### Chromosomal Gradients of Polymorphism and Divergence

**Figure 1:**
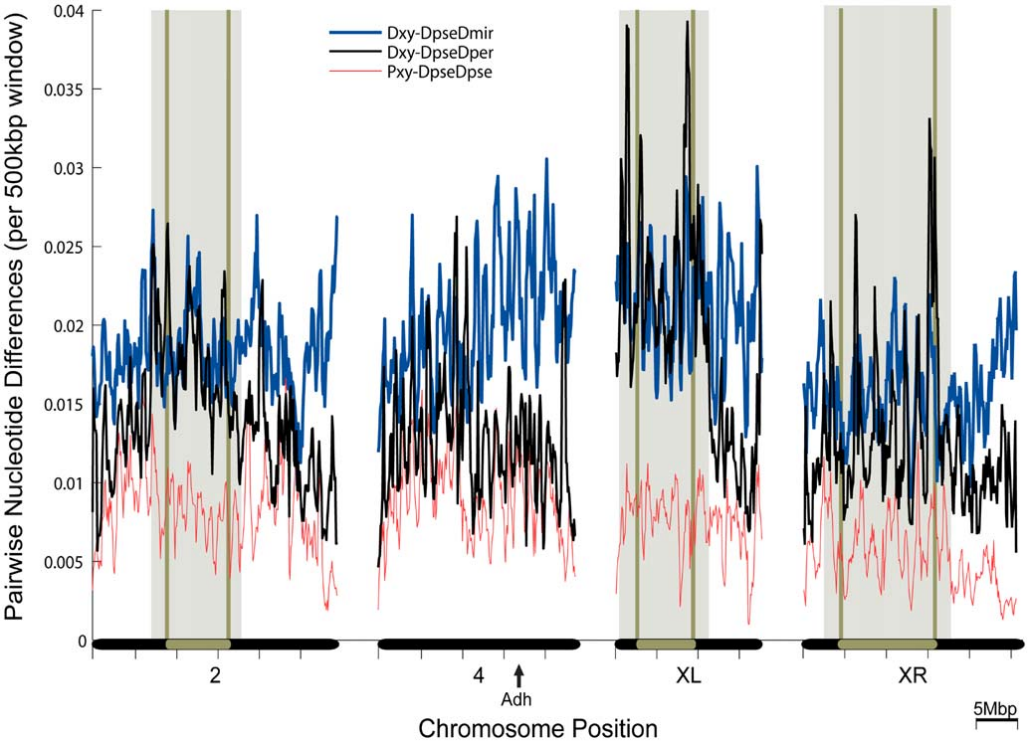
Genome-wide distribution of variation. Divergence (Dxy) between the *D. pseudoobscura* (*Dpse*) and each of *D. persimilis* (*Dper*, black line) and *D. miranda* (*Dmir*, blue line) is estimated in overlapping 500 kbp windows (100 kbp steps) across chromosome arms 2, 4, XL, and XR. All chromosome arms are presented from the telomere to centromere (left-to-right), though the sequence assemblies start well outside both the telomere and centromere in each case. Nucleotide diversity (Pxy) is estimated as the pairwise differences between genomic sequences of two strains of *D. ps. pseudoobscura* (red line). The breakpoints of the inversions distinguishing *D. ps. pseudoobscura* and *D. persimilis* are superimposed on each chromosome (rounded brown rectangle) and on the graph (vertical brown lines). The region where recombination is suppressed in hybrids is boxed in gray. Contigs from chromosomes are oriented and ordered according to previous studies. Each chromosomal increment is 5 Mbp. The positive control locus, *Adh*, is highlighted on chromosome 4 group 1.

Figure 1 presents sliding window estimates of polymorphism within the North American subspecies, *D. ps. pseudoobscura*, and divergence between *D. ps. pseudoobscura* and each of *D. persimilis* and *D. miranda* across four of the five major chromosome arms. Each datapoint within the sliding window represents the fraction of bases differentiating two genome sequences along a 500 kilobase pairs (kbp) interval, iterated every 100 kbp. Very similar plots were generated for intergenic regions or introns alone (not shown). We only scored positions for which aligned sequences were available for all four taxa (Table S2), hence eliminating the possibility that a particular region of high or low divergence would be represented in some estimates but not others. Nucleotide polymorphism estimated within *D. ps. pseudoobscura* was confirmed to be in the same range as that observed in polymorphism studies of focal genomic regions of this species [18] (see Table S3).

Fixed inversions on chromosomes XL, XR, and 2 distinguish *D. ps. pseudoobscura* and *D. persimilis*, and their breakpoints are superimposed on Figure 1. Corroborating previous work, nucleotide diversity within *D. ps. pseudoobscura* and divergence between *D. ps. pseudoobscura* and *D. persimilis* are low in regions near the centromere [21],[29]. The latter observation was previously interpreted “as reflective of ancestral patterns of polymorphism rather than the process of divergence between these species” [21]. Consistent with this interpretation, we observe that diversity within *D. ps. pseudoobscura* and divergence between *D. ps. pseudoobscura* and *D. persimilis* were correlated on every chromosome arm (r = 0.418–0.535, *P*<0.01 for each) [see also 24].

The species pair, *D. ps. pseudoobscura* and *D. miranda*, exhibit a different pattern. There was no consistent decline in divergence between these two species in regions near the centromeres. Furthermore, diversity within *D. ps. pseudoobscura* was not significantly correlated with divergence to *D. miranda* along any chromosome arm except chromosome 4 (r = 0.330, *P* = 0.018), suggesting that *D. miranda* and *D. ps. pseudoobscura* are not sharing many polymorphisms. According to a model where *D. miranda* is the outgroup, we predict that the range (maximum minus minimum) of divergences across windows should be greater for the purportedly more divergent species pair, *D. miranda - D. ps. pseudoobscura*, than the pair of more recently diverged species, *D. persimilis - D. ps. pseudoobscura*. While this prediction was met for windows along the collinear chromosome 4, we observed instead a greater range of divergences in the *D. persimilis - D. ps. pseudoobscura* pairing on the chromosome arms (XL, XR, and 2) that harbor inversions distinguishing these species (see also Table S4, Figure S1). These observations are inconsistent with a more recent divergence of this latter species pair, and are more consistent with the presence of multiple genealogical histories along the genome.

### Recombination and Divergence around Inversion Breakpoints

Inversions prevent gene exchange because the products of recombination are not recovered. We confirmed that recombinant products are not recovered within 2.1 megabase pairs (Mbp) of fixed inversions along chromosome XL, XR, and 2 in heterozygotes (*D. ps. pseudoobscura* - *D. persimilis* interspecies hybrids). We recovered 0.25%–0.55% recombinants at markers 2.8 Mbp outside of each inversion, indicating that complete recombination suppression extends greater than 2.1 Mbp, but not more than 2.8 Mbp outside inversions. Strong crossover suppression, resulting in less than one percent recombinants, is observed relative to one marker 3.35 Mbp outside of the XR chromosomal inversion. Crossing over is largely restored at 4.55 Mbp outside inversions, with a crossover rate greater than 5% observed from one marker on chromosome 2 (see Table S5).

The lack of recombination and introgression should produce a distinct signature in nucleotide divergence within and near chromosomal inversions. We found that, along the three chromosome arms bearing inversions, nucleotide diversity within *D. ps. pseudoobscura* was comparable to *D. persimilis* nucleotide divergence when estimated on sequence greater than 2.5 Mbp outside the inverted regions. In contrast, divergence between *D. persimilis* and *D. ps. pseudoobscura* was comparable to divergence between *D. miranda* and *D. pseudoobscura* in regions inside and within 2.5 Mbp flanking the inversions. The consistency of this pattern across independent chromosomal arms suggests either that all three inversions arose at approximately the same time as the split from the ancestor of *D. miranda*, or that the ancestral populations of these species were already separated (i.e., allopatric) when the inversions arose (see Discussion).

### Recent Autosomal Gene Exchange between *D. ps. pseudoobscura* and *D. persimilis*

Recent gene flow is not expected between the South American *D. ps. bogotana* and either of the North American taxa *D. ps. pseudoobscura* or *D. persimilis*. Analyses of nucleotide sequence data suggests that the *D. pseudoobscura* subspecies diverged from a common ancestor 200,000 years ago [19],[30]. As such, we can use the isolated subspecies as a “negative control” to test for recent introgression between North American *D. ps. pseudoobscura* and *D. persimilis*.

**Figure 2:**
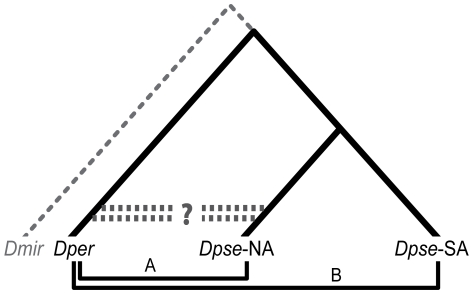
Divergence and gene flow between *D. persimilis* and *D. ps. pseudoobscura*. Genic introgression between the two North American sister species, *D. persimilis* (*Dper*) and *D. ps. pseudoobscura* (*Dpse*-NA), was assayed by comparison to the non-hybridizing isolated South American subspecies, *D. ps. bogotana* (*Dpse*-SA). Recent genic introgression between *Dper* and *Dpse*-NA is expected to generate different levels of divergence (*Dper-Dpse*-NA<*Dper-Dpse*-SA) while an absence of gene flow is expected to produce equivalent levels of divergence (*Dper-Dpse*-NA = *Dper-Dpse*-SA). Differences in lineage-specific evolutionary rates were assessed using a nonhybridizing species outgroup, *D. miranda* (*Dmir*).

Because of hybridization between the North American taxa, a very simple expectation is that *D. persimilis* (*Dper*) should be more similar in sequence to North American (NA) than South American (SA) subspecies of *D. pseudoobscura* (Figure 2). We limited the dataset to sites where we have 454/Roche sequence reads for both *D. pseudoobscura* subspecies, and tested this hypothesis using regions far from the inversion on chromosome 2 and all along collinear chromosome 4. Aligned bases were categorized as [*Dper* = NA≠SA] or [*Dper* = SA≠NA]. No two bases were scored that were within 500 bp of each other, hence reducing artifacts from non-recombining haplotype blocks. We observed an excess of the first category (7073 vs. 6797, Binomial Sign Test *P* = 0.0096), indicating that divergence is lower between *D. persimilis* and North American *D. ps. pseudoobscura* than between *D. persimilis* and South American *D. ps. bogotana*.

The above test does not account for possible faster divergence within the South American subspecies lineage, either through increased mutation rate or more frequent fixation of slightly deleterious alleles. Testing for differences in lineage rates, we did not observe greater divergence between the South American subspecies and the non-hybridizing species, *D. miranda* (*Dmir*), than between the North American subspecies and *D. miranda* (D_xy_ = 0.019 for both, *P* = 0.221). Nonetheless, we can test for recent gene exchange more rigorously by specifically counting “shared-derived” base pair substitutions polarized with *D. miranda*. Counts of [*Dmir* = SA≠*Dper* = NA] were compared to counts of individual base pairs in which [*Dmir* = NA≠*Dper* = SA], where the latter half of the inequality denotes potential shared-derived bases. Again, we observe a slight, borderline significant excess of the first category (219 vs. 185, Binomial Sign test *P* = 0.05), suggesting that *D. persimilis* and North American *D. ps. pseudoobscura* share more derived bases.

Finally, introgression between species is not expected to be homogeneous outside inverted regions. The Alcohol dehydrogenase (*Adh*) region has been reported to have introgressed recently between these species using analyses independent of divergence from the South American species, *D. ps. bogotana* [19]. Further, it can be introgressed in the laboratory and made homozygous in a foreign genetic background with no deleterious effects [23]. We examined base pair counts of [*Dmir* = SA≠*Dper* = NA] vs. [*Dmir* = NA≠*Dper* = SA] for this region. In this region which bears *Adh* (chromosome 4 “group1”, extending 4 Mbp starting at position 14.4 million in Figure 1), we again observed a significant and dramatic excess of the first category (27 vs. 10, *P* = 0.00382).

### Lack of Recent X-Chromosomal Gene Exchange between *D. ps. pseudoobscura* and *D. persimilis*

We applied the same analyses to test for recent gene exchange along X-linked regions from both XL and XR distant from the inversion breakpoints. We observed a nonsignificant difference in number of bases categorized as [*Dper* = NA≠SA] vs. [*Dper* = SA≠NA] on this chromosome (1200 vs. 1131, Binomial Sign Test *P* = 0.079). When we polarized the bases and compared (*Dmir* = SA≠*Dper* = NA) vs. (*Dmir* = NA≠*Dper* = SA), we observed a nonsignificant difference *opposite* in direction to our expectation (46 vs. 62). However, there was only 27% as much sequence to analyze more than 2.5 Mbp from inversions on the X-chromosome than on the autosomes.

## Discussion

Genome sequencing has recently become affordable for individual investigators, but how the resultant data can be applied to address evolutionary questions about species formation or diversification has been less clear. Here, we use partial genome sequence data to: 1) evaluate the role of chromosomal inversions in maintaining the distinction between two hybridizing Drosophila species, 2) estimate when gene exchange occurred between these species, and 3) clarify contradictory interpretations from earlier studies that attempted to address related questions. Overall, this research demonstrates how present-day patterns within genomic data can help to infer past processes involved in speciation.

### Patterns of Differentiation between Species and a Model for Their Divergence

If two species share extensive polymorphism through introgression or incomplete lineage sorting resulting from a recent split, we predict that nucleotide sequence diversity within species should be correlated with average pairwise nucleotide differences between species. Extensive polymorphism sharing was shown previously in the case of *D. ps. pseudoobscura* and *D. persimilis* [18],[20],[21],[31]. In contrast, we find that nucleotide sequence differences between *D. ps. pseudoobscura* and *D. miranda* were uncorrelated with nucleotide sequence differences between two strains of *D. ps. pseudoobscura*. This finding suggests that our comparisons to *D. miranda* are not hindered by introgression or extensive shared ancestral polymorphisms [but see 32].

Previous DNA sequence-based studies observed that *D. ps. pseudoobscura* and *D. persimilis* share variation far outside the fixed inversions that distinguish these species [20]. Here, we note that divergence between *D. ps. pseudoobscura* and *D. persimilis* is higher and more comparable to differences between two strains of *D. ps. pseudoobscura* in regions distant from the inversions. In contrast, divergence between *D. ps. pseudoobscura* and *D. persimilis* is comparable to that between *D. ps. pseudoobscura* and the non-hybridizing outgroup, *D. miranda*, for regions inside and just outside the chromosomal inversions that separate them.

These new results can be used to formulate a hypothesis for the evolutionary history of these species and reconcile previously contradictory inferences. Machado et al [20] suggested that *D. ps. pseudoobscura* and *D. persimilis* largely speciated in allopatry, close in time to the split of these species from *D. miranda*, and recent secondary contact between the first two resulted in the dissolution of differences outside the inverted regions. In contrast, Noor et al [21] noted that significant differences in divergence among the XL, XR, and 2-chromosome inverted regions suggest instead that *D. ps. pseudoobscura* and *D. persimilis* speciated under a sympatric “divergence-with-gene-flow” model. In other words, differences in divergence between inversions reveal when each inversion arose as both species evolved in sympatry.

Our study recapitulates both sets of results and allows us to suggest a resolution. As in Noor et al [21], we observe that the XL chromosome arm inversion was most different in sequence between *D. ps. pseudoobscura* and *D. persimilis*, followed by chromosome 2 and finally chromosome arm XR (see Figure 1). However, we also observe that divergence between *D. pseudoobscura* and *D. persimilis* within each inverted region was similar to the divergence between *D. ps. pseudoobscura* and *D. miranda* (shown at single loci by [20]). Because we observe the same XL>2>XR ranking in *D. ps. pseudoobscura* divergence from *D. miranda* that was shown previously for divergence from *D. persimilis* [21], we now interpret this variation among chromosomes as reflective of differences in mutational processes rather than differences in time since separation. Our new, combined observations suggest two possible interpretations. First, the three inversions independently may have arisen very close in time (near the time of the split from *D. miranda*) from the *D. pseudoobscura-D. persimilis* ancestor, and these three derived forms segregated exclusively in *D. persimilis*. Alternatively, and arguably more parsimoniously, the three species diverged close in time, *D. persimilis* acquired three new inversions sometime after the split from *D. pseudoobscura*, and secondary contact between *D. persimilis* and *D. pseudoobscura* homogenized the noninverted regions.

### Timing of Introgression between Species

Many recent studies have analyzed DNA sequence polymorphism and divergence to identify the statistical signature of recent introgression. However, these tests did not typically identify a time frame within which introgression occurred except as variance in the time of divergence [25],[26]. Instead, most tests merely reject or fail to reject a model of divergence in total isolation.

Here, we use a comparison between subspecies to infer the timing of introgression between *D. persimilis* and *D. ps. pseudoobscura*. One *D. pseudoobscura* subspecies co-occurs and hybridizes with *D. persimilis* while the other subspecies lives isolated on a different continent. Hence, we can attribute differences in divergence between *D. persimilis* and these *D. pseudoobscura* subspecies to hybridization that has occurred more recently than the split of the subspecies, estimated to have been 200,000 years ago [19],[30]. We observe a slight but statistically significant difference in divergence across uninverted (collinear) autosomal regions between *D. persimilis* and the two *D. pseudoobscura* subspecies, suggesting recent introgression between the co-occurring taxa, but we fail to detect such evidence for introgression across comparable regions of the X-chromosome.

Although we detected a statistically significant signature of introgression along autosomal loci, the signature was faint, suggesting that recent gene exchange has not been extensive. DNA sequence-based studies previously identified the statistical signature of historical introgression [18],[20],[31], but these studies interpreted this gene exchange as ancient based on the lack of longer shared haplotypes [33]. Similarly, an allozyme-based meta-analysis failed to detect differences between *D. ps. pseudoobscura* populations co-occurring with *D. persimilis* compared to those elsewhere in North America [34], suggesting a lack of extensive recent introgression. Given the high levels of gene exchange among populations within *D. ps. pseudoobscura*, the approach used by Kulathinal and Singh [24] does not have enough resolution to detect the low levels of gene flux we infer here. Again, our sparse genomic sequence data helps to refine these earlier results.

A significant difference between sex-linked and autosomal loci in introgression has been a recurring theme in divergence population genetics [e.g.], [ 35], [36]–[38]. However, in most systems, we lack knowledge of the karyotype (e.g., inversion differences) or other factors which may make the sex chromosomes and particular autosomes inappropriate for comparison. In *D. ps. pseudoobscura* and *D. persimilis*, however, we observe evidence for introgression on the autosomes while not on the X-chromosome in regions outside the inversions, suggesting that these differences may be reflective of sex-linkage *per se*. This observation may be consistent with a higher density of factors conferring hybrid sterility or other barriers to gene flow on the X-chromosome than on the autosomes [e.g., 39].

### Use of Whole Genome Shotgun Sequences to Infer Processes of Speciation

In this study, we used sparse whole-genome shotgun sequences from multiple taxa to infer the evolutionary history of a species group and to identify genomic features associated with their divergence. Our system was well-leveraged in that we initially began the investigation already having an assembled and annotated full-genome sequence for two of the focal species [12],[28] as well as genetic mapping data localizing factors that reduced potential gene exchange [22],[23].

Nonetheless, the cost of next-generation sequencing is dropping for both model and non-model systems, even between the execution of this study and its publication. Because of cost constraints, our study approached these questions using light resequencing (effectively utilizing the power of millions of markers) but producing extensive gaps and a majority of aligned positions being covered by single sequence traces. However, our approach serves as a proof-of-principle for future genomic studies on lesser developed systems. We attempted to reduce systematic biases by applying stringent filters, specific tests (including averaging across 500 kbp windows) and by employing the use of a well-assembled reference genome sequence. Future, more rigorous approaches enabled by less-expensive sequencing technologies will allow researchers greater power to infer historical evolutionary processes such as speciation and historical introgression in non-model systems.

## Materials and Methods

### Sampled Genomes

In this comparative study, a total of five genomes representing four species of the obscura subgroup were sampled. Adult females from inbred lines of *D. miranda* (from Mather, California; San Diego stock #14011-0101.08) and the subspecies, *D. ps. bogotana* (from El Recreo, Colombia; San Diego stock #14011-0121.152) were each extracted and purified using the Gentra PureGene DNA isolation kit. For *D. miranda*, genomic DNA was nebulized and single stranded libraries generated before being sequenced at light coverage on a single Roche/454 Life Sciences GS-FLX run at Duke University's IGSP core sequencing facility, yielding approximately 100 Mbp of sequence (see Table S1). *D. ps. bogotana* genomic DNA was similarly sequenced in one half of one run at Duke University's IGSP core sequencing facility and one half of one run at 454 Life Sciences. These genome sequence traces were submitted to the NCBI Short Read Archive (SRA) as accession SRA008268. Additionally, two previously sequenced and assembled genomes, *D. ps. pseudoobscura* (Release 2) and *D. persimilis* (Release 1), were used for comparative analysis [12],[28]. Finally, to estimate nucleotide diversity within *D. ps. pseudoobscura*, previously sequenced Roche/454 reads (NCBI SRA accession SRA000268) from a second line (from Flagstaff, Arizona; San Diego stock number 14011-0121.151; [24]) were reassembled syntenically to *D. ps. pseudoobscura*.

### Genomic Assembly, Alignment, and Annotation

All Roche/454 reads were syntenically aligned against reference *D. ps. pseudoobscura* (Release 2) linkage groups. Individual base calls were filtered to exclude nucleotides that are: within 3 base pairs of an alignment gap, harbor low quality scores (below 10), contain greater than 30% mismatches within a 7 base pair window, are in regions of high divergence (divergence to *D. persimilis* is greater than 30% in a 7 base pair window). Alignments from the two previously sequenced reference genomes, *Drosophila ps. pseudoobscura* and *D. persimilis* were obtained via chain files from the UCSC Genome Browser (genome.ucsc.edu). Site-specific annotation information such as intron and codon position was extracted from *D. ps. pseudoobscura* Release 2.3 annotations from FlyBase (flybase.org). Chromosome arms (including ordered contigs) 2, 4, XL, and XR were used (see [40] for contig details), representing roughly 80% of the total genome. We did not survey chromosome 3 because of complications from its inversion polymorphism within each of these species [41].

### Chromosomal Inversions and Recombinational Mapping in the Hybrids

Chromosome arms XL, XR, and 2 differ by single inversions between *D. pseudoobscura* and *D. persimilis*, and the breakpoints of these inversions have been mapped [21],[42]. Using microsatellite markers that flank the sides of each inversion, we surveyed the extent of recombination in F_1_ hybrids between *D. ps. pseudoobscura* and *D. persimilis*. The published genome lines of both species (San Diego stock numbers #14011-0121.94 and #14011-0111.49) were used in this cross and recombinants were screened among 384 progeny of F_1_ females backcrossed to *D. pseudoobscura*. The following markers were used to assay recombination rate at varying distances from the inversions – chromosome 2 inversion: DPS2019 (2.77 Mbp from inversion on telomeric side), DPS2026 (associated with inversion) and DPS2031 (2.8 Mbp from inversion on centromeric side), XL inversion: DPSX_7446z (2.84 Mbp from inversion on centromeric side), DPSX046 (associated with inversion), DPSX008 (0.4 Mbp from inversion on telomeric side), and DPSXL_3a_0.8 (2.8 Mbp from inversion on telomeric side), XR inversion: DPSXR_6_2.7 (3.35 Mbp from inversion on centromeric side), DPSX063 (associated with inversion), DPSX037nA3 (1.4 Mbp from inversion on telomeric side), DPSX037N (2.1 Mbp from inversion on telomeric side), and DPSX058 (2.8 Mbp from inversion on telomeric side). Primer sequences are available upon request.

## Supporting Information

Figure S1 Ratio of divergence to polymorphism (Dxy/Pxy), and differences in divergence over 500 kbp windows. Inversion breakpoints for chromosomes 2, XL, and XR are shown in green. Polymorphism was estimated by comparing genomic sequences between two strains of *D. ps. pseudoobscura*. Divergence estimates were then divided by diversity, the former of which was measured between *D. ps. pseudoobscura* and each of *D. persimils* (red) and *D. miranda* (black). The difference in overall divergence between *D. miranda* and *D. persimilis* against *D. ps. pseudoobscura* is found in grey.
(0.11 MB TIF)

Table S1 “Short-read” sequences used in this study.
(0.02 MB DOC)

Table S2 Aligned and filtered nucleotides common to four species. The total aligned sequence per chromosome with scored bases for all of the following four samples: *D. pseudoobscura* (published genome), *D. pseudoobscura* (Flagstaff, AZ), *D. persimilis* (published genome), and *D. miranda* (Mather, CA). This common set of base pairs were used in Figure 1 and other associated analyses.
(0.02 MB DOC)

Table S3 Comparison of *D. ps. pseudoobscura* nucleotide diversity across studies. Estimates of nucleotide diversity from *D. ps. pseudoobscura* from our study (focusing on intergenic regions) were compared to a previous study of nucleotide diversity in focal intergenic regions (Machado et al. 2002). The results show that roughly the same range of values of diversity were recovered from the two works (0.0026–0.0210 vs 0.0024–0.0179).
(0.03 MB DOC)

Table S4 Percent difference from *D. pseudoobscura* across 400 kbp windows. Numbers indicate lowest and highest percent differences across windows for each chromosome arm separately, as well as the observed range (highest-lowest).
(0.02 MB DOC)

Table S5 Markers surveyed to examine recombination fractions in hybrids for various points at known physical distances from the inverted regions. For each chromosome, markers are all listed from the centromere to telomere, including the marker within the inversion. Physical distances from each marker to the nearest inversion breakpoint and the recombination fractions observed from the marker within the inversion are also indicated.
(0.03 MB DOC)
