# Supplementary figures and images for: The Genomics of Speciation in Drosophila: Diversity, Divergence, and Introgression Estimated Using Low-Coverage Genome Sequencing
Source: PLoS Genet. 2009 Jul 3;5(7):e1000550. doi: 10.1371/journal.pgen.1000550 (PMC2696600; doi:10.1371/journal.pgen.1000550)

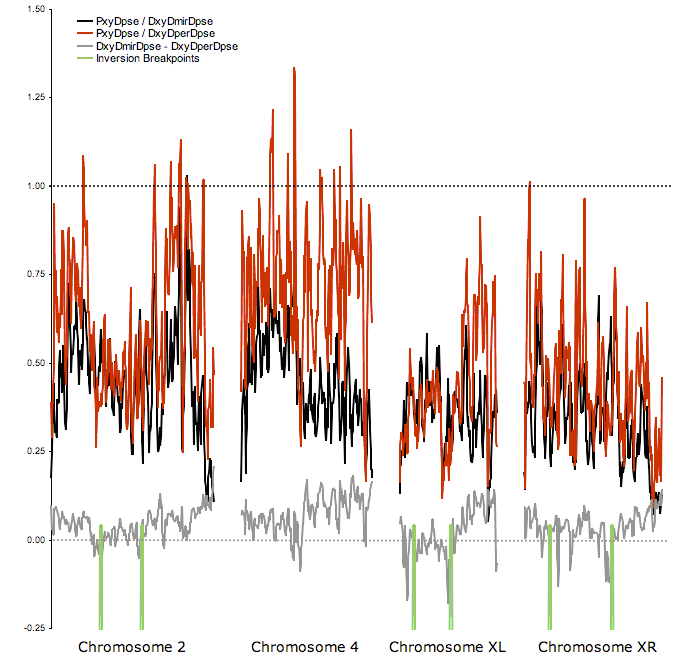

Supplement: Figure S1 — Ratio of divergence to polymorphism (Dxy/Pxy), and differences in divergence over 500 kbp windows. Inversion breakpoints for chromosomes 2, XL, and XR are shown in green. Polymorphism was estimated by comparing genomic sequences between two strains of D. ps. pseudoobscura. Divergence estimates were then divided by diversity, the former of which was measured between D. ps. pseudoobscura and each of D. persimils (red) and D. miranda (black). The difference in overall divergence between D. miranda and D. persimilis against D. ps. pseudoobscura is found in grey. (0.11 MB TIF) [file pgen.1000550.s001.tif]
