# Supplementary material for: The Genomics of Speciation in Drosophila: Diversity, Divergence, and Introgression Estimated Using Low-Coverage Genome Sequencing
Source: PLoS Genet. 2009 Jul 3;5(7):e1000550. doi: 10.1371/journal.pgen.1000550 (PMC2696600; doi:10.1371/journal.pgen.1000550)
Supplement: Table S1 — “Short-read” sequences used in this study. (0.02 MB DOC) [file pgen.1000550.s002.doc]

**Table S1.** “Short-read” sequences used in this study.

Sample Total Sequence Obtained NCBI SRA Accession

*D. miranda* 110.0 Mbp SRX003254

(Mather, CA)

*D. pseudoobscura bogotana* 80.3 Mbp SRX003252, SRX003253

(El-Recreo, Colombia)

*D. pseudoobscura pseudoobscura* 66.1 Mbp SRX001087

(Flagstaff, AZ)
