# Supplementary material for: The Genomics of Speciation in Drosophila: Diversity, Divergence, and Introgression Estimated Using Low-Coverage Genome Sequencing
Source: PLoS Genet. 2009 Jul 3;5(7):e1000550. doi: 10.1371/journal.pgen.1000550 (PMC2696600; doi:10.1371/journal.pgen.1000550)
Supplement: Table S2 — Aligned and filtered nucleotides common to four species. The total aligned sequence per chromosome with scored bases for all of the following four samples: D. pseudoobscura (published genome), D. pseudoobscura (Flagstaff, AZ), D. persimilis (published genome), and D. miranda (Mather, CA). This common set of base pairs were used in Figure 1 and other associated analyses. (0.02 MB DOC) [file pgen.1000550.s003.doc]

**Table S2.** Aligned and filtered nucleotides common to four species. The total aligned sequence per chromosome with scored bases for all of the following four samples: *D. pseudoobscura* (published genome), *D. pseudoobscura* (Flagstaff, AZ), *D. persimilis* (published genome), and *D. miranda* (Mather, CA). This common set of base pairs were used in Figure 1 and other associated analyses.

Chromosome Aligned/Filtered Base Pairs

XL 817,250

XR 1,408,930

2 1,550,675

4 1,221,618
