# Supplementary material for: The Genomics of Speciation in Drosophila: Diversity, Divergence, and Introgression Estimated Using Low-Coverage Genome Sequencing
Source: PLoS Genet. 2009 Jul 3;5(7):e1000550. doi: 10.1371/journal.pgen.1000550 (PMC2696600; doi:10.1371/journal.pgen.1000550)
Supplement: Table S3 — Comparison of D. ps. pseudoobscura nucleotide diversity across studies. Estimates of nucleotide diversity from D. ps. pseudoobscura from our study (focusing on intergenic regions) were compared to a previous study of nucleotide diversity in focal intergenic regions (Machado et al. 2002). The results show that roughly the same range of values of diversity were recovered from the two works (0.0026–0.0210 vs 0.0024–0.0179). (0.03 MB DOC) [file pgen.1000550.s004.doc]

**Table S3.** Comparison of *D. ps. pseudoobscura* nucleotide diversity across studies. Estimates of nucleotide diversity from *D. ps. pseudoobscura* from our study (focusing on intergenic regions) were compared to a previous study of nucleotide diversity in focal intergenic regions (Machado *et al*. 2002). The results show that roughly the same range of values of diversity were recovered from the two works (0.0026-0.0210 vs 0.0024-0.0179).*

Previous Estimated Diversity Present Study Estimated Diversity

Locus (Machado *et al.* 2002) (Intergenic bps in 500kbp region)

XL: DPSX008 0.0210 0.0093

XR: DPSX009 0.0139 0.0076

XR: DPSX010 0.0026 0.0024

2: DPS2001 0.0108 0.0162

2: DPS2002 0.0152 0.0179

2: DPS2003 0.0068 0.0103

4: DPS4002 0.0026 0.0067

4: DPS4003 0.0194 0.0072

*Note: We are not concerned with the differences between the results of our study and that of Machado *et al.* (2002) for specific regions. Our study took an average diversity from sparse sampling of two strains across a 500,000 bp window while Machado *et al.* (2002) examined 600-1000 bp within these regions in 15-20 strains.
