# Supplementary material for: The Genomics of Speciation in Drosophila: Diversity, Divergence, and Introgression Estimated Using Low-Coverage Genome Sequencing
Source: PLoS Genet. 2009 Jul 3;5(7):e1000550. doi: 10.1371/journal.pgen.1000550 (PMC2696600; doi:10.1371/journal.pgen.1000550)
Supplement: Table S4 — Percent difference from D. pseudoobscura across 400 kbp windows. Numbers indicate lowest and highest percent differences across windows for each chromosome arm separately, as well as the observed range (highest-lowest). (0.02 MB DOC) [file pgen.1000550.s005.doc]

**Table S4.** Percent difference from *D. pseudoobscura* across 400 kbp windows. Numbers indicate lowest and highest percent differences across windows for each chromosome arm separately, as well as the observed range (highest-lowest).

Chromosome All sequence Intergenic regions

XL *D. persimilis* 0.00692-0.03928 (0.03236) 0.00687-0.05310 (0.04624)

*D. miranda* 0.01290-0.03008 (0.01719) 0.01389-0.03421 (0.02032)

XR *D. persimilis* 0.00558-0.03314 (0.02756) 0.00310-0.04078 (0.03768)

*D. miranda* 0.00979-0.02334 (0.01354) 0.00688-0.02539 (0.01851)

2 *D.persimilis* 0.00671-0.02514 (0.01843) 0.00554-0.03825 (0.03272)

*D. miranda* 0.01227-0.02731 (0.01504) 0.01122-0.02857 (0.01735)

4 *D. persimilis* 0.00463-0.02072 (0.01608) 0.00391-0.03046 (0.02656)

*D. miranda* 0.01194-0.03056 (0.01862) 0.00507-0.03905 (0.03398)
