# Supplementary material for: The Genomics of Speciation in Drosophila: Diversity, Divergence, and Introgression Estimated Using Low-Coverage Genome Sequencing
Source: PLoS Genet. 2009 Jul 3;5(7):e1000550. doi: 10.1371/journal.pgen.1000550 (PMC2696600; doi:10.1371/journal.pgen.1000550)
Supplement: Table S5 — Markers surveyed to examine recombination fractions in hybrids for various points at known physical distances from the inverted regions. For each chromosome, markers are all listed from the centromere to telomere, including the marker within the inversion. Physical distances from each marker to the nearest inversion breakpoint and the recombination fractions observed from the marker within the inversion are also indicated. (0.03 MB DOC) [file pgen.1000550.s006.doc]

**Table S5.** Markers surveyed to examine recombination fractions in hybrids for various points at known physical distances from the inverted regions. For each chromosome, markers are all listed from the centromere to telomere, including the marker within the inversion. Physical distances from each marker to the nearest inversion breakpoint and the recombination fractions observed from the marker within the inversion are also indicated.

_________________________________________________________________________________________

Chr Marker/Pos/Recomb <Inversion> Marker/Pos/Recomb Marker/Pos/Recomb Marker/Pos/Recomb

**_________________________________________________________________________________________**

XL x7446z <DPSX046> DPSX008 XL_3a_0.8*

2.84MB 0.4MB 2.8MB

1/380 0/379 1/380

XR XR6_2.7* <DPSX063> DPSX037NA3 DPSX037N DPSX058

3.35MB 1.4MB 2.1MB 2.8MB

3/364 0/372 0/368 2/361

2 DPS2031mod* <DPS2026> DPS2019

4.55MB 2.8MB

19/360 1/373

**_________________________________________________________________________________________**
